# Reactive transport modelling of an intra-basalt sandstone reservoir, Rosebank, UK

**DOI:** 10.1038/s41598-021-86421-w

**Published:** 2021-03-25

**Authors:** C. Sætre, H. Hellevang, C. Dennehy, H. Dypvik

**Affiliations:** 1 Department of Geosciences, University of Oslo, Blindern, P.O box 1047, 0316 Oslo, Norway; 2 Norwegian Geotechnical Institute (NGI), Torgarden, PO Box 5687, 7485 Trondheim, Norway; 3 The University Centre in Svalbard (UNIS), Pb. 156, 9171 Longyearbyen, Norway; 4 Equinor UK Ltd, Equinor House, Prime Four Business Park, Kingswells, AB51 8GQ Aberdeen UK

**Keywords:** Solid Earth sciences, Geochemistry, Geology, Mineralogy, Petrology, Volcanology

## Abstract

The Rosebank field, located in the Faroe-Shetland Basin, contains producible hydrocarbons in intra-basaltic siliciclastic reservoirs. The volcanic-reservoir interface is poorly studied and the geochemical system, as a function of distance from the basalt, is largely unknown. The current paper applies a geochemical model coupling mineral dissolution and precipitation with element diffusion to investigate the geochemical system in time and space from the basalt-sandstone interface. Earlier studies indicate few negative effects on reservoir properties despite the proximity to a reactive volcanic lithology. The causes of this minimal impact have not been studied. The numerical simulations in this study expand on the knowledge demonstrating that precipitation of authigenic phases at the basalt-sandstone interface buffer the formation water solution for key elements, which hamper the transport of solutes and subsequent precipitation of secondary minerals within the reservoir. Saturation index values over the simulated period indicate that precipitation of authigenic phases should not extend beyond the basalt-sandstone interface. This shows that diffusion alone is not enough to reduce the reservoir quality due to increased precipitation of secondary phases. The basalt dissolution rate varies according to the silica concentration. The combined effects on silica concentration by diffusional fluxes, mineral precipitation and dissolution, control the basalt dissolution rate, and there are no differences in the results between high and low basalt reactive surface area.

## Introduction

Sandstones containing volcaniclastic material often have poor reservoir quality. This is due to the early development of secondary mineral phases and mechanical compaction reducing permeability and porosity^1,2^. The early formation of secondary mineral phases is a result of the high reactivity of the volcanic detritus^3^. Basalts on Rosebank contain both crystalline phases and glasses of basaltic composition. Both crystalline phases and glasses have a high dissolution rate compared to silica rich minerals, however glasses have a higher dissolution rate compared to the crystalline equivalent^4^. The Rosebank field was discovered in 2004 and proved a new play concept in the region with producible reservoirs in intra and sub-volcanic reservoirs^5^. There are several places around the world where hydrocarbons are produced from volcaniclastics or volcanics, e.g. around Nuussuaq in the West Greenland^6,7^, the Samgori‐Patardzeuli and Muradkhanly fields in the Caucasus^8^, Liaohe Basin in China^9^, the Yurihara field in Japan^10^, and in the Cambay Basin in India^11^.

**Figure 1:**
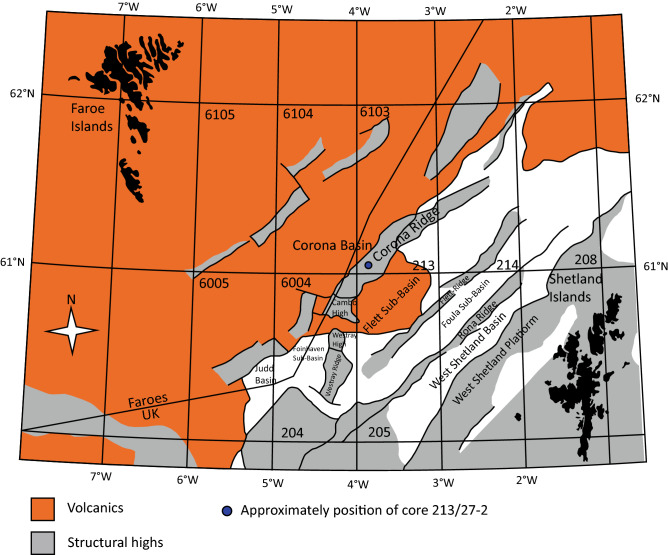
Overview of the Faroe-Shetland Basin (FSB) with approximately position of core 213/27-2. Modified from^13^.

The Rosebank field is situated on the UK Continental Shelf within the Faroe-Shetland Basin (FSB) (Fig. 1). It is operated by Equinor UK Ltd (40%) with Suncor Energy (40%) and Siccar Point Energy (20%) as coventurers.

Fluvio-deltaic reservoir sandstones of the Flett Fm. are found as intra-volcanic siliciclastic reservoir sandstones^5^ where the reservoir sand thickness varies over the throughout the Rosebank area^15,16^. The volcanic lithologies consist of extrusive basalts and volcaniclastics^5^, whereas the siliciclastic sandstones stand in contrast as “clean” with minimal input of mafic minerals. This was likely a result of the source rocks being situated outside the volcanic areas^12,13^. Incorporation of unstable minerals may reduce reservoir properties due to precipitation of pore-filling secondary mineral phases^2,3,14^. This, however, does not seem to be the case in the Rosebank Field, and the result is an excellent reservoir with permeabilities up to Darcy range^14–16^.

The present study builds upon the work done by^16^ and ^15^ who studied the diagenetic system of the Rosebank reservoirs. These studies demonstrated that reservoir properties seemed unaffected by the close spatial proximity of mafic lithologies. This seems mainly due to limited incorporation of mafic minerals in the siliciclastic reservoir sands and little to no diffusive transport of elements from the basaltic lavas into the reservoirs. The net effect is a highly effective reservoir where the basalts did not promote precipitation of clay minerals in the siliciclastic sandstones. The boundary between the basalts and reservoir sandstones are, however, poorly studied, and the geochemical system in this area is largely unknown.

The current paper intends to explore the effects of having volcanic lithologies within the petroleum system. This study uses the PHREEQC v3^17^ geochemical model coupling basalt dissolution, precipitation of authigenic phases and element diffusion to demonstrate how far basalt dissolution and element transport may affect the reservoir sands. The intention is to increase the understanding of how this system works with an emphasis on reservoir quality.

## Geological setting

The Faroe-Shetland Basin (FSB) is located between the Faroe Islands and the Shetland Isles (Fig. 1). It consists of series of NE–SW-trending sub basins separated by Mesozoic and Paleozoic highs with Rosebank located on the southern part of the Corona ridge (Fig. 1)^5,18^.

The FSB developed through a series of rifting and compressional events from the Paleozoic to Cenozoic. This resulted in the development of several NE-SW sub-basins of the FSB (Fig. 1)^18–20^. The main hydrocarbon source on Rosebank is assumed to be the Kimmeridge Clay Formation^21,22^. Volcanic activity in the Late Paleocene-Early Eocene resulted in thick flood basalt accumulations^23^. Simultaneously, the fluvial to shallow marine siliciclastic reservoir sandstones on Rosebank were deposited^5,12,13,24^. This system competed for accommodation space with locally sourced basalts resulting in the interstratified basalts and sandstones of the Flett Fm.^12,13^. Studies by^2^ and ^3^suggests that the sedimentary system extends around the Cambo High and prograded NE along the Corona Ridge (Fig. 1).

Compressional events in the Oligocene–Miocene resulted in localized uplift of variable magnitude in the Faroe-Shetland region [e.g. ^25,26^]. For Rosebank, this resulted in approximately 800 m of uplift^27^. Current reservoir temperatures at Rosebank is about 80 °C while fluid inclusion studies indicate paleotemperatures up to ~ 120 °C^16^. In some locations in the West of Shetland region there are indications of vertical transport of hot fluids e.g.^50,51^. The authors are not aware of any publications that suggests a similar transport in the Rosebank area. Previous studies by^16^ and ^15^ report diagenetic observations that can be explained by a closed isochemical system. Additionally, fluid inclusion temperatures observed in quartz overgrowth can be explained by a deeper burial prior to the Oligocene–Miocene uplift^16^.

## Results

For a detailed description and discussion on authigenic and primary mineral phases observed on Rosebank the reader is referred to^16^ and ^15^.

### Scenario 1: Base case without basalt dissolution

**Figure 2:**
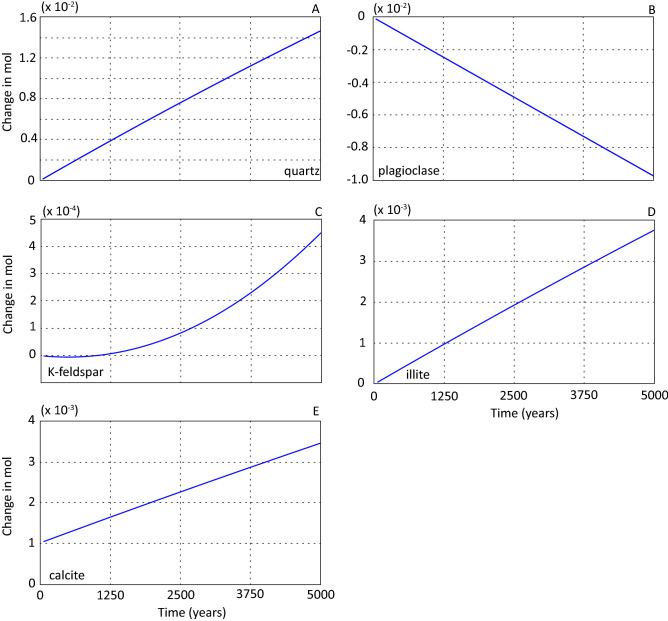
Temporal development of mineral abundances for primary (**A**–**C**) and secondary (**D**–**E**) minerals presented for cell #31 over the simulated period for scenario 1. All results are displayed as change in moles from the starting amount.

**Figure 3:**
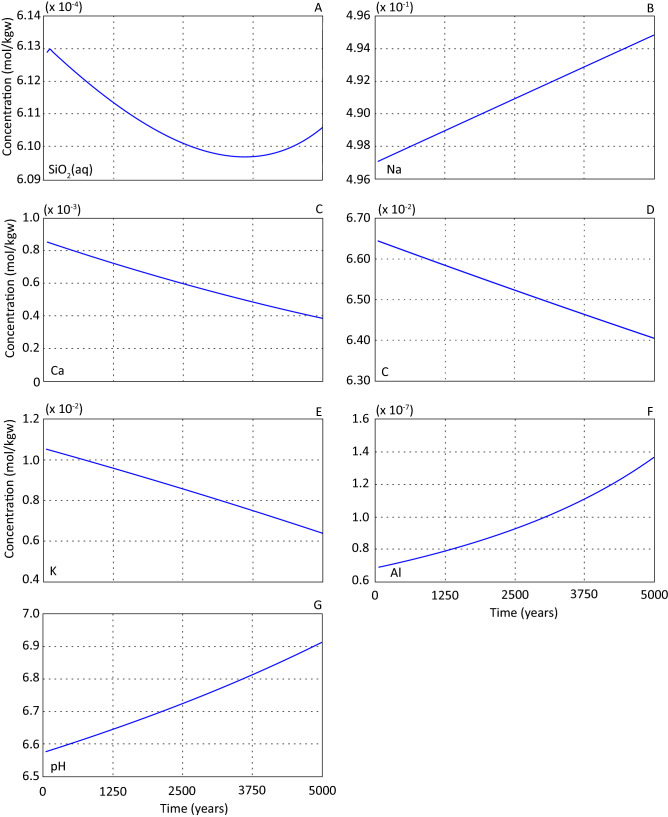
Element concentration (mol/kg water) and pH presented for cell #31 over the simulated period for scenario 1.

Figures 2 and 3 illustrate the temporal changes in mineral abundances and element concentrations for a sandstone and formation water 77.5 cm from the basalt interface (cell #31) over the simulated period. There is no basalt dissolution in this scenario, and any sandstone cell will be representative for changes in the whole column.

Evolution of primary minerals (Fig. 2A–C) and the initial formation water (Table 3) provided necessary elements for precipitation of secondary minerals (Fig. 2D,E). Quartz reached saturation and precipitated throughout the simulated period (Fig. 2A), and the accompanying silica concentration of formation water was approximately constant throughout the simulated period (Fig. 3A). The change in aqueous silica concentration reflects that supply of silica from plagioclase dissolution (Fig. 2B) exceeds the silica consumption by precipitating quartz (Fig. 2A), K-feldspar (Fig. 2C) and illite (Fig. 2D).

Plagioclase dissolved at a constant rate throughout the simulation (Fig. 2B) showing that changes, due to evolution of primary and secondary minerals, and subsequent formation water element concentrations, did not affect the dissolution rate. After 5000 years of simulation a total of 4.86 × 10^–5^ mol, or 0.13% of the initial plagioclase had dissolved. Contrasting this, K-feldspar precipitated after an initial period of dissolution (Fig. 2C), but the rate of precipitation was significantly lower than plagioclase dissolution rate. Plagioclase dissolution released Ca and Na to the formation water. The sodium concentration (Fig. 3B) increased linearly over the simulated period as no precipitated secondary phases consumed sodium. The Ca concentration, on the other hand, decreased (Fig. 3C), accompanied by a reduction in dissolved carbon (Fig. 3D) due to precipitation of calcite (Fig. 2E).

Precipitation of K-feldspar (Fig. 2C) and illite (Fig. 2D) consumed potassium from the solution resulting in a successive potassium concentration decrease (Fig. 3E). The Al concentration built up during the simulated period (Fig. 3F) due to the dissolution of plagioclase. Precipitation of secondary minerals holding Al (illite and K-feldspar) was not enough to buffer the solution with respect to this element (Fig. 3F). It should be noted that the Al concentrations were low throughout the simulated period (Fig. 3F). Aluminium concentrations were too low for boehmite precipitation which was sub-saturated over the simulated period. Albite saturation increased over the simulated period because of increasing Na and Al concentrations. Due to the lack of precipitation of Mg-containing secondary phases the magnesium concentration remained constant through the modelled period. The pH of the system increased from ~ 6.21 to ~ 6.91 (Fig. 3G) due to silicate mineral water interactions.

### Scenario 2 and 3: Basalt dissolution with varying reactive surface areas

**Figure 4:**
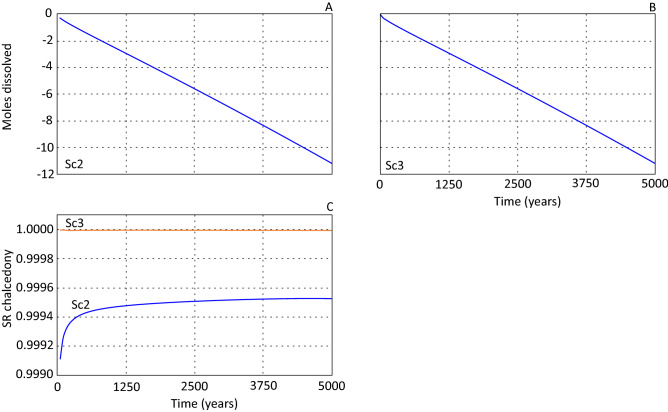
Basalt dissolution displayed for cell #1 (Fig. 2) as change in moles as a function of simulated time for scenario 2 (**A**) and 3 (**B**) where scenario 3 was simulated with a reactive surface area two orders larger than scenario 2. (**C**) shows saturation ratio (SR) for chalcedony in cell #1 for scenario 2 and 3 over the simulated time.

The available reactive basalt surface area differentiated scenario 2 and 3. Scenario 2 was simulated with 0.16 m^2^/kgw while scenario 3 was simulated with 16 m^2^/kgw. The dissolution rate has a first order dependency on available reactive surface area (Eq. 4), and the two scenarios were modelled to assess the sensitivity of the available reactive surface area. The results showed insignificant differences between scenario 2 and 3 in the amount of dissolved basalt over the simulated period (Fig. 4A,B). The explanation for this is the higher silica saturations in scenario 3 compared to 2 (Fig. 4C), where the affinity term in the rate equation counterbalanced the larger surface area. The saturation ratio for chalcedony depends on the silica concentration which was controlled by the diffusional flux of silica and precipitation of Si holding phases.

**Figure 5:**
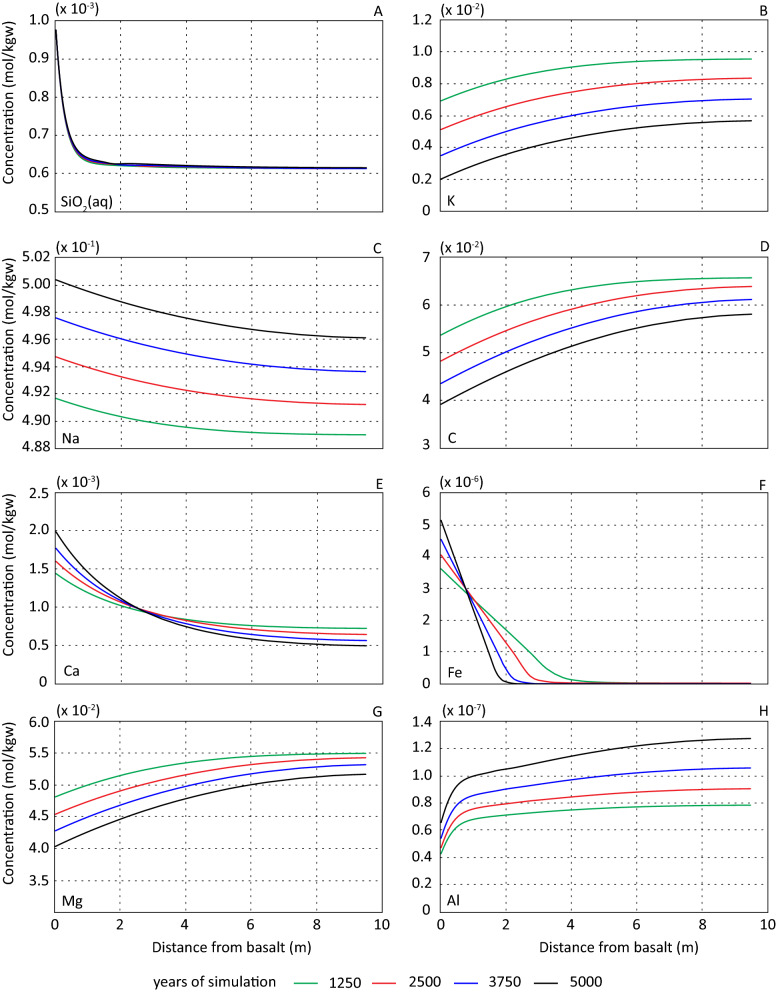
Formation water element concentrations (mol/kgw). Results are displayed for scenario 2 as distance from basalt-sediment interface after 1250, 2500, 3750 and 5000 years of simulation.

As the difference in basalt dissolution between scenario 2 and 3 was insignificant, there were also no differences in the resulting element concentrations and evolution of primary and secondary phases. Results for scenario 2 and 3 are therefore presented together using scenario 2 as an example. All dissolved elements were affected by the basalt and developed various concentration gradients from the basalt-sandstone interface into the sandstone (Fig. 5).

**Figure 6:**
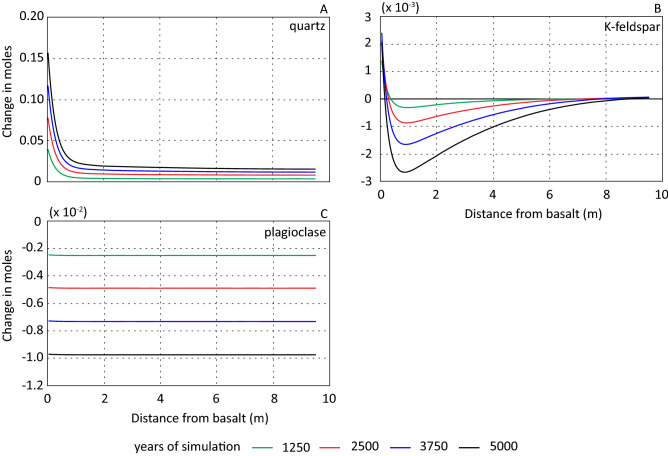
Change in mole from initial amounts for primary minerals. (**A**–**C**) show results with distance from basalt-sandstone interface in meter after 1250 (green), 2500 (red) 3750 (blue) and 5000 (black) years for scenario 2.

Released silica from the dissolving basalt resulted in a steep and stable silica gradient from the basalt-reservoir interface into the sandstone (Fig. 5A). The stable position and concentration magnitude show that a steady state was reached, constrained by the primary and secondary phases and diffusional flux of silica as described above. Furthermore, the amount of precipitated quartz (Fig. 6A) was reflected by the silica concentration gradient (Fig. 5A). The largest increase in quartz (0.18 mol/kgw) occurred in the cell adjacent to the basalt-sandstone interface (Fig. 6A). At the far end of the column (with reference to the basalt-sandstone interface) the amount of precipitated quartz was similar to the base case scenario (Fig. 2A). This limited the increase in quartz precipitation due to the presence of basalt to the first meters (Fig. 6A).

**Figure 7:**
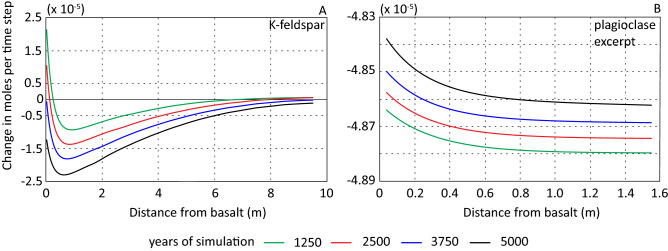
Change in mol (rate) of K-feldspar (**A**) and plagioclase (**B**) as a function of time and distance from basalt displayed for scenario 2. Note difference in x-scale.

**Figure 8:**
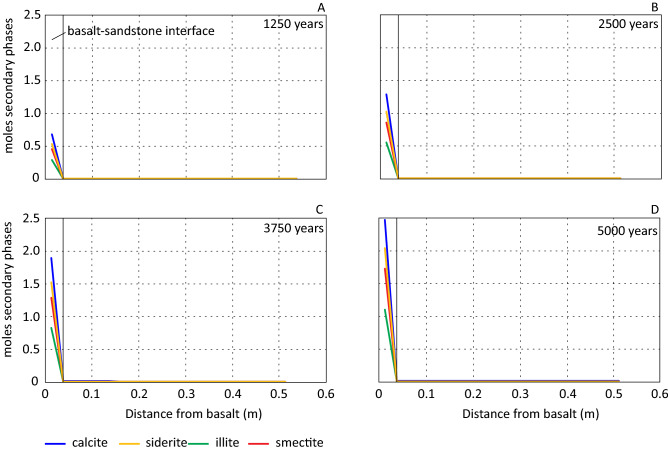
Change in moles of secondary minerals with distance from the basalt-sandstone interface after 1250, 2500, 3750 and 5000 years.

Introducing basalt to the system influenced both the temporal and spatial evolution of K-feldspar (Figs. 6B and 7A). In scenario 1 there was a small increase in K-feldspar over the simulated period (Fig. 2C). In scenario 2 and 3 K-feldspar precipitated in cells closest to the basalt-sandstone interface and at a distance of > 7 m the first approximately 3000 years of simulation (Fig. 7A). The highest precipitation rate occurred in the adjacent cell to the basalt-sandstone interface and the rate rapidly decreased with distance from the basalt. After ~ 3000 years of simulation, K-feldspar dissolved throughout the column with a maximum dissolution rate at about 1 m from the basalt-sandstone interface (Fig. 7A). At the end of the simulation, the amount of K-feldspar in the adjacent cells to the basalt was still higher than at the start of simulation while the abundance in the remaining column was lower (Fig. 6B). The temporal and spatial evolution of K-feldspar may be due to competing affinity for potassium between illite and K-feldspar. In excess of 1 mol/kgw illite precipitated at the basalt-sandstone interface at end of simulation (Fig. 8). The resulting potassium gradient dipping towards the basalt (Fig. 5B) was a result of more potassium being consumed by illite precipitation than released by basalt and K-feldspar dissolution.

Generally, plagioclase was minimally affected by the presence of the basalt (Fig. 6C) compared to the base case scenario, but there was a small retardation in the dissolution rate close to the basalt-sandstone interface (Fig. 7B). The difference was small, and the formation waters was far from equilibrium with respect to plagioclase for most of the simulated column. The dissolution of plagioclase did release Ca and Na to the solution, where the former was incorporated in precipitated calcite (Fig. 8). No secondary phases consumed sodium, and the concentration increased over the simulation with additional sodium released from the basalt (Fig. 5C).

The carbon content in the formation water decreased progressively over the simulated period and developed a concentration gradient towards the basalt-sandstone interface (Fig. 5D). Precipitation of carbonate minerals consumed carbon, and the resulting concentration gradient reflected the increased precipitation of calcite and siderite at the basalt-sandstone interface (Fig. 8). The amount of precipitated calcite per time step was not enough to buffer the total released calcium from basalt and plagioclase. This resulted in increasing Ca concentration over the simulated period from the basalt and up to approximately 3 m (Fig. 5E). At greater distances Ca concentration was progressively reduced due to calcite precipitation consuming more Ca than made available by basalt and plagioclase dissolution.

Beside illite, smectite (saponite) was the only clay mineral formed over the modelled period, and the occurrence was mainly limited to the basalt-sandstone interface (Fig. 8). Simulated formation waters were undersaturated with respect to nontronite throughout the simulated period without a significant increase in saturation indices. Due to the reducing conditions iron was mainly in the ferrous state with ferric concentrations below nontronite saturation levels and iron was consumed by precipitation of smectite and siderite (Fig. 8). The basalt dissolution rate decreased over the first simulated steps observed by increased chalcedony saturations (Fig. 4C). The initial higher dissolution rate resulted in the diffusion of Fe throughout the column, but the concentration was low at a distance > 4 m (Fig. 5F). The consequence of iron diffusion through the column was precipitation of a small amount of smectite (maximum c. 4.0 × 10^–4^ mol) outside the basalt-sandstone interface. After the initial formation the amount of smectite precipitated per time step progressively decreased without precipitation of significant amounts at the end of the simulation. Over the simulated period, the iron gradient became steeper and retreated towards the basalt-sandstone contact as a result of precipitation of iron bearing phases. At end of simulation the iron concentration at > 2.5 m was < 1.05 × 10^–9^ mol/kgw.

Precipitation of smectite consumed Mg from the formation water resulting in a continuous decrease in Mg concentration (Fig. 5G). The amount of smectite formed at the basalt-sandstone contact (Fig. 8) consumed more Mg than released from the dissolving basalt putting up a concentration gradient towards the basalt. The same holds for Al (Fig. 5E) consumed by precipitation of illite and smectite (Fig. 8) and released by dissolving feldspars and basalt.

**Figure 9:**
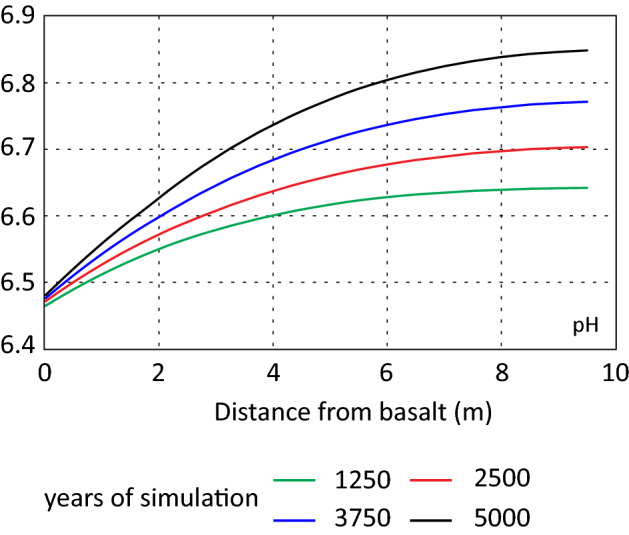
Temporal and spatial evolution of pH.

Figure 9 shows the spatial and temporal pH evolution. At the far end of the column the pH development was like the base case scenario. Close to the basalt the increase in pH was smaller which most likely reflects the interaction of the dissolving basalt and precipitation of authigenic phases resulting in a more stable pH.

**Figure 10:**
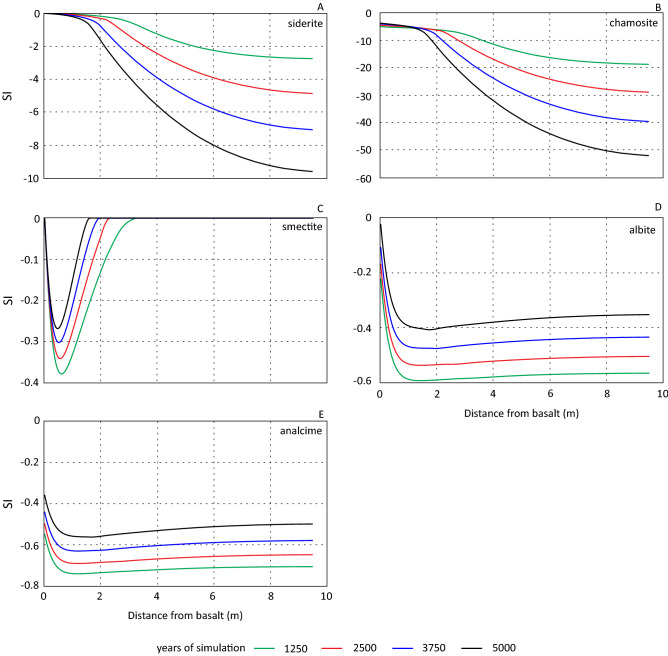
Temporal and spatial evolution of saturation index (SI) siderite (**A**), chlorite (chamosite) (**B**), smectite (**C**), albite (**D**) and analcime (**E**).

Precipitation of authigenic phases due to the presence of the basalt (scenario 2 and 3) occurred mainly at the basalt-sandstone interface. Additionally, this area did not expand over the simulated period (Fig. 8). Figure 10 displays saturation index (SI) values for some secondary minerals. The figure illustrates that formation of siderite (Fig. 10A) and chlorite (chamosite) (Fig. 10B) may not occur further into the reservoir with increased simulation time. Siderite became progressively more undersaturated in the reservoir section over time, and chlorite (chamosite) was undersaturated throughout the modelled column. Smectite forms instead of siderite and chlorite (Fig. 10C). It reached saturation within the column, and a small amount precipitated due to the diffusion of Fe as described above. The dip in SI values close to the basalt may be a result of lowered Mg concentrations preventing an increase in smectite SI values. The general trend of smectite SI values indicates the precipitation of smectites outside the basalt-sandstone contact may not occur with increased simulation time.

Albite and analcime progressed towards saturation over the modelled period (Fig. 10D,E) and might reach saturation over a longer simulated period. The higher SI values close to the basalt probably reflects the additional sodium released from the basalt.

## Discussion

### Model evaluation

The choice of primary and secondary minerals was based on petrographic studies of reservoir and basalt sections^16^ and should give a good background to assess the effect of introducing a basaltic lithology to the system. There are uncertainties in the choice of secondary phases as clay minerals have wide compositional variations where thermodynamic and kinetic data are not available for all compositions. Applying a few representative clay mineral phases where thermodynamic data is available should, however, be an acceptable approximation and addition of other clay species to the model should not change the overall trends. Kaolinite occurs in some Rosebank core samples but was omitted in the model. The occurrence of kaolinite on Rosebank was interpreted as an early diagenetic phase formed at the expense of feldspars and/or mica leached by meteoric water [e.g.^28,29^]. Due to the low solubility of aluminium, illite and boehmite were selected as Al-bearing phases allowed to precipitate to avoid unreasonable build-up of aluminium concentration in formation waters.

Precipitation of secondary phases in the numerical simulations sourced necessary elements from the initial formation water and from leached elements by dissolving primary minerals and basalt. Key elements such as aluminium and iron were not present in the initial formation water, and the availability of these was controlled by the dissolution of primary minerals and basalt. The rate of dissolution of these was in turn controlled by kinetic rate equations. Consequently, the kinetic rate equations used controlled the amounts of key elements available for secondary phases.

The reactive basalt surface area for scenario 2 (0.16 m^2^/kgw) was calculated based on the total available surface area of the basalt-sandstone interface assuming no connected basalt pore space. This is presumed a realistic lower reactive surface area for a coherent basalt. The high reactive surface area used in scenario 3 was assigned 100 times larger than scenario 2 in order to investigate the impact of increased reactive surface area. The difference in dissolution rate was insignificant between the modelled scenarios as the chalcedony saturation index controlled the basalt dissolution rate. The use of chalcedony in the affinity term of basaltic glass is supported by a silica-enriched layer forming at the basalt-water interface, and the dissolution rate can therefore be simulated as a function of solution silica concentration and distance from amorphous silica equilibrium^30^.

The selection of diffusion coefficient might have an important control on precipitation of secondary phases in the reservoir section. If the value used was unrealistically low, then key elements (e.g. silica, iron and magnesium) could be transported out of the basalt-sandstone contact at a faster rate than simulated. This might promote precipitation of Mg and Fe containing minerals (smectites, siderite) within the sandstone reservoir. Additionally, a higher diffusion rate could have resulted in faster basalt dissolution rates due to increased silica diffusive flux. The value chosen is assumed to be a best approximation by applying known diffusion rates and incorporating tortuosity in the calculation. The value is most likely conservative as it does not consider porosity reduction due to precipitation of pore filling secondary phases. A reduction in porosity would increase the tortuosity^31^ and ultimately reduce the diffusion coefficient. Precipitation of pore lining illite is a known permeability reducing process which was not considered by the model [e.g.^32–34^].

The temporal and spatial development of pH is an uncertainty irrespective of scenario. This is inferred as pH in sedimentary basins is commonly buffered by metastable equilibrium between fluid and minerals (carbonates, sulfides, metal oxides and aluminium silicate minerals)^35,36^, and basalt-water interactions should produce higher pH than observed^36^. This may indicate that the simulated system did not attain equilibrium with respect to a pH buffer system within the simulated period. If albite reaches saturation over a longer simulated period, this can result in a mineral assemblage of larger buffer capacity^36^. Additionally, formation of albite might reduce aluminium concentrations that showed a small increase throughout the simulated period. The observed pH difference from the basalt-sandstone contact into the sandstone may be a result of the larger amount of precipitating clays at the contact consuming H^+^.

Carbonate minerals precipitated throughout the simulation and would progress if carbon is available in formation waters. When carbonate precipitation is reduced, or halted Ca and Fe would be available for other secondary minerals, and a subsequent increase in precipitation of smectites could be expected.

The simulated period was fairly short but considered long enough to assess the general trends of introducing a basalt to the system. Increased precipitation of secondary phases (clays and carbonates) over a longer simulated period seems unlikely assessing temporal and spatial development of saturation indexes (SI). This is assumed as SI values for siderite and chlorite did not progress towards saturation further into the reservoir than the basalt-sandstone contact. Smectite is an exception as it reached saturation within the sandstone, but the amount formed was small and controlled by iron availability. The simulations illustrate retraction of the iron concentration gradient towards the basalt with increased simulation time. This was due to buffering of the element by precipitation of iron bearing secondary phases at the basalt-sandstone interface. It seems unlikely that an increased simulation period should result in significant amounts of iron diffusing into the sandstone.

The model is conceptual and considering the identified uncertainties the simulations seem to meet and answer the main question of the work, to assess the effects of having a reactive mafic lithology within the reservoir section.

#### Comparison with Rosebank studies and effects on reservoir properties

The simulations predict increased formation of secondary phases (clay minerals and carbonates) only at the basalt-sandstone contact. In general, these phases consumed elements released from the basalt resulting in element concentration gradients indicating limited diffusion into the sandstone. This is in-line with bulk XRF analysis of Rosebank samples where no observable increase in e.g. FeO_2_ towards the basalts was observed^16^. The formation water composition on Rosebank is unknown, but if it contains e.g. elevated iron concentrations it is not reflected in the bulk rock chemistry. In the sandstone cells adjacent to the basalt there was an increase in quartz precipitation and K-feldspar dissolution. Assessing the difference in molar volumes of quartz (22.68 cm^3^/mol) and K-feldspar (108.87 cm^3^/mol) from the carbfix database^37^ it is reasonable to believe that there should not be a net loss of porosity due to the increased precipitation of quartz.

The results of the simulations and petrographic studies of the Rosebank reservoir and basalt units compare well. Quartz precipitated during the simulation and may be analogous to quartz overgrowths observed in the Rosebank samples. Formation of authigenic quartz is reasonable, taking the temperature into account. Quartz overgrowths are typically associated with temperatures > 75 °C [e.g.^38^]. The silica source is however different in natural systems where the source is normally assumed to be local and provided by quartz dissolution along stylolites and clay laminae^39,40^. In contrast, the silica source in the simulations seems to be plagioclase dissolution. After 5000 years of simulation 0.13% of the plagioclase had dissolved. Assuming a constant dissolution rate it would take approximately 3.66 million years to dissolve all plagioclase. However, plagioclase reacted after a kinetic rate equation taking the available surface area into account. The surface area was calculated based on the initial mol of the mineral present, see Eqs. 6 and 10 in the method chapter. The time before all plagioclase had dissolved should therefore be significantly longer than 3.66 million years.

Authigenic clay minerals observed in the Rosebank sandstone samples (chlorite coatings and smectite/illite) were interpreted as an alteration product of detrital phases^16^. Formation of chlorite coatings in the Rosebank sandstones seems likely considering the shallow marine to fluvial depositional environment^41^. The absence of chlorite in the simulated sandstone section is reasonable, as in natural systems they are commonly interpreted to be a result of alteration of an iron or magnesium rich precursor coating phase^41–45^ which is absent in the model. Similarly, observed smectite/illite on Rosebank may have formed as an alteration product from detrital smectite^16^. The precipitated illite in the simulation may serve as an analogue to this as a sink for aluminium and potassium, equivalent to smectite-illite alteration^46,47^. It should be noted that it is not suggested that the mechanism for illite and quartz precipitation, as observed in the model, is a natural occurring process.

The cored basalt section in well 213/27-2 consisted of several authigenic phases: carbonates, smectite-chlorite and analcime^16^. These phases were interpreted as secondary phases caused by basalt-water interactions and compare well with the results of the simulations. Analcime was incorporated in the model, but it was undersaturated throughout the simulated period but progressed towards equilibrium.

## Summary and conclusions

A 1D reactive transport model has been applied to explore the geochemical system between siliciclastic reservoir rocks and an adjacent basalt, equivalent to the Rosebank field. Former diagenetic studies of the Rosebank reservoir rocks have not analyzed the basalt-sandstone contact, which has been the main target of the present study, displaying a minimal effect on the basalt-sandstone interface.

Basalt dissolution results in increased precipitation of smectite and carbonates only at the basalt-sandstone interface. This buffered the solution for key elements (e.g. Mg and Fe) preventing formation of large amounts of authigenic minerals within the reservoir section. Silica concentrations and thereby the chalcedony saturation index (SI) was controlled by the combination of diffusive flux and dissolution/precipitation of primary and secondary phases. This resulted in insignificant differences of basalt dissolution rates irrespective of available reactive basalt area. By assessing the temporal and spatial development of mineral SI values it seems unlikely that a prolonged simulation period would result in a significantly different result.

## Methods

### Numerical model

For modelling of dissolution, precipitation and diffusion the PHREEQC (v3) model was used^17^. The model couple mineral–water interactions and diffusion, providing an evaluation of mineral precipitation and dissolution with respect to both distance from the basalt-interface and time. The model was built to reflect Rosebank reservoir conditions and core 213/27-2 was used as a basis for primary and secondary minerals, as well as basalt chemistry^16^.

**Figure 11:**
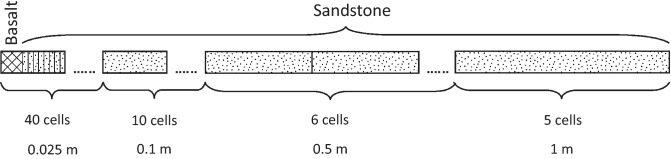
Conceptual representation of the 10-m 1D column modelled over a period of 5000 years with dissolution, precipitation and diffusion. The column was divided into 61 cells with the following size distribution from left to right: 40 cells of 0.025 m, 10 cells of 0.1 m, 6 cells of 0.5 m and 5 cells of 1 m. The first cell contained basalt of tholeiitic composition in scenario 2 and 3 while the remaining cells contain detrital minerals according to Table 1. In scenario 1 all cells contained detrital minerals as presented in Table 1.

Figure 11 illustrates a conceptual drawing of the one-dimensional (1D) diffusion model. The simulations are intended to reflect the intra-basaltic siliciclastic reservoir setting of the Rosebank Field. In this case the reservoir sands are enclosed by basalts and reservoir-edges are in contact with a basalt layer. The model mimics this by simulating geochemical reactions in a column where one end represents the basalt layer and the remaining column represents the reservoir sand. Simulated interactions at the basalt-reservoir system would be relevant all intra-basaltic reservoir sections. In-depth descriptions of the system can be found in previously published literature [e.g.^5,12,13,15,16^].

A closed system was assumed only allowing diffusional transport of solutes^48,49^. Diffusion was simulated for a column discretized into 61 cells with basalt located in the first cell and the remaining cells representing the sandstone section (Fig. 11). Cell dimension increased progressively from the first cell to the column end having a total column length of 10 m representing the permeable sandstone sandwiched between two tight basalt layers. Boundary conditions for the first and last cells were of Neumann type (closed)^17^. The justification of column length is twofold. Increased column length requires longer computational time. In addition, in previous studies^16^ shale intervals were reported within the sandstones. These intervals should function as baffles for fluid flow.

Former studies have proposed vertical transport of hot fluids in some locations in the West of Shetland region [e.g.^50,51^]. This has not been observed proposed for the Rosebank field or the Corona high. Studies by^16^ argues for a closed system by observations of a set of diagenetic features that can be explained by a closed isochemical system at observed temperatures.

In the simulations a fixed diffusion coefficient was applied to all elements, the multicomponent diffusion capability of PHREEQC^17^ was not utilized. The multicomponent diffusion may have resulted in small differences in diffusion coefficients for the solutes. Small differences in diffusion coefficients are however assumed to be subordinate for the purpose of this paper.

### Modelled scenarios

Three different scenarios were modelled, each over a period of 5000 years. The timespan is short in the context of natural slow diagenetic processes but long enough to indicate effects a basaltic lithology may have on reservoir properties. Scenario 1 represents the diagenetic system in the absence of basalt and is used as a base case to evaluate the effect of introducing a mafic lithology to the system. The base case scenario (1) was equilibrated with respect to formation of common authigenic phases.

### Selection of primary and secondary phases, and water composition

Primary and secondary phases used in the model were based on petrographic studies of the Rosebank core samples^16^. Basalt chemistry was adopted from^16^ (Table 1). Minerals assumed to form by basalt-water reactions but auto-formed in the sandstone were suppressed by forcing oversaturation by fixing a SI of 3 for saponite-Mg–Fe (Table 1) for precipitation. Quartz and feldspar amounts were averaged for core 213/27-2 in the depth range between 2932.32 to 2953.38 mRKB^16^. Some modifications of secondary phases were necessary, as thermodynamic data for intermixed smectite/chlorites phases were not available. To accommodate this, several different smectite and chlorite compositions were used (Table 1). Boehmite (AlO(OH)) and illite were incorporated as secondary phases to avoid build-up of Al concentrations to unreasonable levels.

**Table 1:** Initial abundance of primary and secondary phases applied to the model.

	Initial abundance (XRD%)	^1^LogK^0^
Primary minerals (chemical formula)		
**Sandstone**		
^2^Quartz (SiO_2_)	67	–
^2^ K-feldspar (KAlSi_3_O_8_)	10	–
^2^Plagioclase (Ca_0.2_Na_0.8_Al_1.2_Si_2.8_O_8_)	23	–
**Basalt**		
^3^Basalt (Na_0.096_K_0.003_Fe_0.210_Mg_0.217_Al_0.350_Ca_0.268_Si_1.000_O_3.379_)	100	–
**Secondary minerals**		
Calcite (CaCO_3_)	0	1.8487
Siderite (FeCO_3_)	0	− 11.0441
Amorphous SiO_2_ (SiO_2_)	0	− 2.7136
Chamosite (Fe_5_Al_2_Si_3_O_10_(OH)_8_)	0	32.8416
Saponite-Mg-Fe (Fe_0.175_Mg_3_Al_0.35_Si_3.65_O_10_(OH)_2_)	0	27.6789
Nontronite-Mg (Mg_0.175_Fe_2_Al_0.35_Si_3.65_H_2_O_12_)	0	− 11.6200
Illite (KAl_3_Si_3_O_10_(OH)_2_)	0	13.5858
Analcime (Na_0.96_Al_0.96_Si_2.04_O_6_:1H_2_O)	0	− 15.4836
Boehmite (AlO(OH))	0	7.5642
Albite (NaAlSi_3_O_8_)	0	2.7645

^1^All thermodynamic data comes from the carbfix.dat database^37^. 0 note standard state (temperature of 298 K and 1 bar pressure).

^2^Initial abundance normalized for quartz, K-feldspar and plagioclase.

^3^Basalt chemistry from^16^.

Primary siliciclastic minerals and basalt reacted according to kinetic rate equations as stated below, while secondary phases precipitated and dissolved based on the principle of local thermodynamic equilibria^52^. The choice of using a local thermodynamic equilibrium for the secondary phases is to reduce the complexity of the model and reduce computation time. Additionally, this is a common assumption due to the low solubility of aqueous aluminium and the aluminosilicates^41^. Mineral abundances and kinetic parameters used are displayed in Tables 1 and 2, respectively. Incorporated minerals were available through the carbfix.dat thermodynamic database^37^.

**Table 2:** Parameters used for modelling of primary minerals.

Parameters	Quartz	K-feldspar	^1^Oligoclase	^2^Rosebank basalt
${k}_{acid}^{298.15}$(mol/m^2^s)	–	8.71 × 10^–11^	30.46 × 10^–12^	3.16 × 10^–6^
${k}_{neutral}^{298.15}$(mol/m^2^s)	1.02 × 10^–14^	3.89 × 10^–13^	–	–
${k}_{basic}^{298.15}$(mol/m^2^s)	5.13 × 10^–17^	6.31 × 10^–22^	2.73 × 10^–12^	7.94 × 10^–12^
Ea_acid_ (kJ/mol)	–	51.7	–	25.5
Ea_neutral_ (kJ/mol)	87.7	38.0	–	–
Ea_basic_ (kJ/mol)	87.7	94.1	–	83.8
n^acid^	–	0.5	–	0.70
n^basic^	− 0.5	− 0.823	–	− 0.33
Molar mass (g/mol)	60.08	278.33	265.42	121.3
β (m^2^/g)	0.0121	0.0125	0.0121	0.16/16

Data for quartz and K-feldspar were taken from^63^. Rate equation data for oligoclase was taken from^65^ and is stated in the Theoretical background.

^1^See theoretical background text for rate equation parameters.

^2^Calculated based on^63^.

Solutes from a seawater standard was used^53^ to simulate mineral interaction and diffusion. The initial composition was modified by equilibrating the solution at reservoir temperature with quartz, K-feldspar and calcite. The carbon content was calculated reflecting calcite equilibrium and CO_2_ fugacity reported for North Sea hydrocarbon reservoirs at 80 °C which equals log pCO_2_ of 0.07^35^. Finally, redox potential was calculated based on hematite-magnetite equilibrium at reservoir temperature^54^ giving a pE of − 4.261 (Table 3). This was done as the formation water composition at Rosebank is unknown. The resulting fluid composition is displayed in Table 3. PHREEQC use an extended Debye-Hückel (Truesdell-Jones) model to simulate the ion activity^17^.

**Table 3:** Fluid compositions for initial seawater.

Component	^1^Initial
	mol/kgw
C	6.749 × 10^–2^
Ca	1.891 × 10^–3^
Cl	5.657 × 10^–1^
K	1.058 × 10^–2^
Mg	5.506 × 10^–2^
Na	4.870 × 10^–1^
SiO_2_	5.746 × 10^–4^
pH	6.21
pe	− 4.261

^1^Modified from^53^.

### Diffusion

Diffusion was modelled using an effective diffusion coefficient *D*_*eff*_ calculated from:

1 $${D}_{eff}=\frac{{D}_{aq}}{{\tau }_{f}}$$

where *D*_*aq*_ is the diffusion coefficient (m^2^/s) in pure water for an arbitrary element and *τ*_*f*_ is the tortuosity factor. The tortuosity factor was related to porosity according to^31^:

2 $${\tau }_{f}={\phi }^{-1.2}$$

The element diffusion coefficient depends on temperature and to a lesser degree on pressure^55^. The increase in the diffusion coefficient can be calculated according to^56^:

3 $${D}_{aq, T}=\frac{{D}_{aq, 298K}T{n}_{298}}{\left(298{n}_{T}\right)}$$

where *T* is the absolute temperature at the investigated conditions and *n* is the temperature dependent water viscosity. At a temperature of 25 °C *n* approximate 1.3 × 10^–9^ m^2^/s. Assuming Rosebank parameters with a porosity of 25%, reservoir temperature of 80 °C, and viscosity from^57^ an effective diffusion coefficient of 1.9025 × 10^–10^ was calculated applying Eqs. (1) to (3). The effective diffusion coefficient was constant for all solutes throughout the simulation and transport of solutes were only modelled by diffusion. The justification of this is that there are no indications of fluid flow on Rosebank and all diagenetic phases observed can be explained by a closed isochemical system^16^. Despite that transport of hot fluids have been proposed in several locations in the West of Shetland region [e.g.^50,51^], temperatures on Rosebank can be explained by a deeper burial during the Oligocene–Miocene^16^. Changes in diffusion coefficient was not considered in the simulations. Over millions of years clogging of pore space may increase the tortuosity resulting in a lower diffusion coefficient. The net result would be a smaller impact on the reservoir by the presence of a basalt. The choice of having a constant diffusional coefficient for all solutes throughout the simulations would be a conservative approach.

### Rate equations for primary minerals and basalt

Rate equations for basalt, quartz and K-feldspar were simulated using a simplified transition-state-theory rate law considering pH and distance from equilibrium^58,59^. For a phase *m*:

4 $${r}_{m}={S}_{m}{k\left(T\right)}_{\left(m\right)}{a}^{n}\left(1-\Omega \right)$$

*r*_*m*_ is the dissolution rate (mol/s), *S*_*m*_ is the reactive surface (m^2^), *k(T)* is the temperature dependent specific rate coefficient (mol/m^2^s), *a* is the proton (H^+^) activity, *n* is the reaction order with respect to H^+^, and *Ω* is the saturation state:

5 $$\Omega =exp\left(\frac{{\Delta G}_{r}}{RT}\right)= \frac{Q}{K}$$

where *∆G*_*r*_ is the Gibbs free energy of reaction, *R* is the gas constant, *T* is the absolute temperature, and *Q* and *K* are the ion activity product and equilibrium constant, respectively.

Reaction rates depend on the available reactive surface area (*Sm*) and calculated according to^60^:

6 $${S}_{m}=\beta MnX$$

*β* (m^2^/g) is the specific surface area, *M* is the molar weight (g/mol), *n* is mole and *X* is the fraction of the total surface area that is reactive at a given time. *β* values for primary silicate minerals were calculated assuming a geometric surface as Brunauer-Emmet-Teller (BET) surfaces were not available for Rosebank sands. This approximation assumes that the sand consists of equal spherical grains with radius *r* (m) and density *ρ* (g/m^3^):

7 $$\beta = \frac{3}{\rho r}$$

For the reservoir sand, an average grain size of fine sand was used, within the range of grain sizes in Rosebank siliciclastic sands^16^. The reactive surface area is uncertain and has been suggested to vary by several orders^61,62^, with old and aged surfaces being at the lower end. Therefore, all minerals were assigned a fraction of reactive surface sites X of 0.001.

Three scenarios were modelled. Scenario 1, without a basalt, functions as a reference scenario, and two scenarios (2 and 3) distinguished by basalt reactive surface area. The available reactive surface area is unknown, but it has a major effect on the rate of basalt dissolution.

The lava flow was assumed to have no connected porosity, and only the interface between the basalt and sand was reactive. The total interface area of the basalt-sediment was estimated based on the geometry of a model cell according to:

8 $${b^2} = \, 1/a \, \phi$$

where *a* is the length of the grid cell, *ϕ* is sandstone porosity, and *b* is the length of a quadratic section of the basalt-sandstone interface. A sandstone porosity of 25% and the model cell length of 0.25 dm, gives an interface surface area of 0.16 m^2^ per litre water, and this value was used for scenario 2. With a larger part of the basalt being reactive in scenario 3, and to illustrate the sensitivity of the model to the surface area, we increased the surface area by two orders of magnitude for scenario 3.

The temperature dependent reaction rate *k(T)* (Eq. 4) was calculated according to:

9 $${k(T)}_{i}={k}_{i}^{298.15}exp\left(\frac{{-Ea}_{i}}{R}\left(\frac{1}{T}-\frac{1}{298.15}\right)\right)$$

where *Ea* is activation energy (J/mol), *R* is the gas constant (8.3145 J/mol K), *T* is absolute temperature, and *i* denotes the three rate equations at acidic, neutral or basic conditions.

Kinetic parameters for quartz and K-feldspar were adopted from^63^. Basalt rate parameters were calculated by piecewise linear regression from Fig. 9 in^64^, assuming a basaltic glass.

Dissolution of plagioclase feldspar (oligoclase) was simulated using a modified version of the rate equation from^65^:

10 $${r}_{oligoclase}={S}_{m} \left(-{k}_{1}\left(1-exp\left(-n{g}^{m1}\right)\right)-{{k}_{2}\left(1-exp\left(-g\right)\right)}^{m2}\right)$$

where *n*, *m1* and *m2* are dimensionless parameters: 8.4 × 10^–17^, 15.0 and 1.45 respectively. *k*_*1*_ equals 30.46 × 10^–12^, and *k2* is 2.73 × 10^–12^ (mol/m^2^s), *g* is given the thermodynamic driving force. Application of the rate equation from^65^ should provide more precise simulated dissolution rates close to equilibrium. The equation was derived for albite but assumed to be an acceptable approximation for oligoclase.

Plagioclase and basalt should not precipitate under the modelled conditions, and this was achieved by the addition of a logical statement hindering precipitation at a saturation ratio higher or equal to 1.
